# What do black patients expect from orthodontic treatment? The aesthetic perception of facial profile between orthodontists and black laypersons

**DOI:** 10.1590/2177-6709.27.4.e2220519.oar

**Published:** 2022-09-05

**Authors:** Denise Bitencourt de SOUZA, Adriana Ismerim OLIVEIRA, Giovana Renata GOUVÊA, Milton SANTAMARIA-JR

**Affiliations:** 1 Fundação Hermínio Ometto, Programa de Pós-Graduação em Ortodontia (Araras/SP, Brazil).

**Keywords:** Perception, Face, Lip, Chin

## Abstract

**Objective:**

To evaluate the influence of anteroposterior position of the soft tissue on facial attractiveness in black people, and compare the perception of aesthetics and satisfaction between orthodontists and black laypersons.

**Methods:**

The sample was composed of 69 orthodontists and 69 laypersons of black ethnicity (n=138). Facial profile photographs of two black volunteers, a man and a woman, were digitally manipulated to change the position of the lips and chin, by making gradual changes of 4mm in relation to the true vertical line, simulating advance or retrusion of the soft tissues by -2, -6, -10, +2, +6, +10mm, totalling six images per sex. The photographs were classified by the research participants using a visual analogue scale (VAS), from 0 (unpleasant) to 100 (pleasant). The results were analyzed by generalized linear model and by the Fisher’s exact test, considering the level of significance of 5%.

**Results:**

The orthodontists and black laypersons considered straight profiles the most pleasant. The two groups classified the male profile as being more unpleasant in comparison with the female facial profile, which was concave. When evaluating all the images together, the image most indicated as being the most pleasant, once again, was the one with the straight profile, for both sexes.

**Conclusion:**

The influence of orthodontists’ and laypersons’ aesthetic perception on evaluating the facial profile of blacks was similar. The straight profile was classified as the most pleasant and the concave, as the most unpleasant.

## RESUMO

**Objetivo:**

Avaliar a influência do posicionamento anteroposterior dos tecidos moles na atratividade facial de indivíduos negros, e comparar sua agradabilidade entre ortodontistas e leigos.

**Métodos:**

A amostra foi composta por 138 avaliadores, 69 ortodontistas e 69 indivíduos leigos da raça negra. Fotografias de perfil de dois pacientes negros, um homem e uma mulher, foram modificadas quanto ao posicionamento dos lábios e do mento, deslocados digitalmente em incrementos de 4 mm em relação a uma linha vertical verdadeira, simulando avanços e recuos dos tecidos moles de -2, -6, -10, +2, +6, +10 mm, totalizando seis imagens por sexo, classificadas pelos participantes da pesquisa usando uma escala visual analógica (EVA) de 0 (desagradável) a 100 (agradável). Os resultados foram analisados por meio de modelos lineares generalizados, considerando-se que todos os observadores avaliaram todas as imagens. A análise das escolhas das imagens consideradas mais agradáveis ou mais desagradáveis foi realizada pelo teste exato de Fisher, com um nível de significância de 5%.

**Resultados:**

Os ortodontistas e leigos consideraram mais agradáveis as alterações que se aproximavam do perfil reto. Nas imagens em que havia birretrusão facial, os dois grupos classificaram como mais desagradável o perfil do homem do que o perfil da mulher. Ao avaliar todas as imagens juntas, a imagem mais indicada como a mais agradável foi, novamente, a com perfil reto, para os dois sexos.

**Conclusão:**

A influência da percepção estética dos ortodontistas e leigos na avaliação do perfil facial de negros foi semelhante, sendo o perfil reto classificado como o mais agradável e o côncavo, o mais desagradável.

## INTRODUCTION

Orthodontic patients are of different ethnicities and educational levels, and their perception of beauty also differs. From this perspective, in orthodontic treatment, concepts and norms must be adapted in order to avoid a standard outcome of treatment for all patients. Therefore, orthodontists must perceive the patient as a unique being, whose self-esteem after conclusion of the treatment is as important as the technical results achieved.^1^ There is considerable variability in the faces of black people, which differ from those of other races. Thus, it is not suitable to apply the aesthetic standards of soft tissue that have been defined for Caucasian patients. The concept of beauty is subjective, an individual preference, and no racial study can be applicable to all individuals of other races.^2^ Black persons have facial features such as acute nasolabial angle, higher and lower lips protrusion, a more pronounced mentolabial groove, and an increased lip-chin ratio,^3^ when compared with Caucasians.

Therefore, facial profiles can be considered esthetically unpleasant even in the absence of conditions of dental malocclusion. A typical example is bimaxillary protrusion, a characteristic prevalent among black persons. This results in a convexity of the facial profile that is frequently unacceptable, in spite of an Angle Class I with normal overjet and overbite, with well-aligned dental arches.^4^ ^-^ ^6^

The soft tissue and bone structure in black individuals are more protruded, in comparison with the pattern for white individuals. A demand for orthodontic treatment to reduce labial protrusion is common among black patients.^7^ Changes in dentofacial tissues after orthodontic treatment and retrusion of the maxillary and mandibular incisors result in decreased convexity of soft tissues.^8^

Due to the scarcity of aesthetic standards for facial analysis of black persons, in the literature,^9^ ^-^ ^11^ this study was conducted to help orthodontists in the quest to achieve more satisfactory treatment results for black patients. Therefore, the aim of this study was to evaluate the influence of soft tissues anteroposterior position on the facial attractiveness in black people, and compare the perception of aesthetics and satisfaction of orthodontists and black laypersons.

## MATERIAL AND METHODS

### SAMPLE

This study was approved (CAAE 85890418.4.0000.5385) by the Research Ethics Committee of the *Fundação Hermínio Ometto*. The ethnicity of the evaluators was determined by self-declaration. Thus, the sample was composed by Caucasian orthodontists and black laypersons. A pilot study was conducted with 30 evaluators, being 15 orthodontists and 15 black laypersons. The sample size in each group was calculated using the Gpower^12^ and R (R Core Team, 2018) programs, and resulted in a minimal number of 67 orthodontists and 67 black laypersons, with large effect size, *f* =0.50, test power of 0.80 and level of significance of 0.05. Based on these data, the final sample of the study was of 138 evaluators: 69 orthodontists and 69 black laypersons, who signed the term of Free, Prior and Informed Consent (FPIC).

### IMAGE ACQUISITION AND MANIPULATION

Side view photographs were taken of two young adult black patients, one male and one female. The volunteers constituted the instrument of evaluation of the research, and signed an authorization allowing the use of their images. Standardized photographs of the facial profile were taken by an experienced photographer, in a studio (Digital Nikon Camera D7000 16.2 MP, Nikon objective 18-105 mm). 

The facial profile photographs were captured in a seated position,^13^ with the Frankfurt plane parallel to the ground and perpendicular to the axis of the body (natural position), and with the interpupilar line parallel to the ground. The volunteers were placed against a white background at a distance of 1.5 m from the photographic camera.^14^

A True Vertical Line (TVL) was placed perpendicular to the ground and afterwards transferred to a computed image, passing through the subnasal point (Sn). This line was used as reference for the advancement or retrusion of the lips and chin.^15^

**Figure 1:**
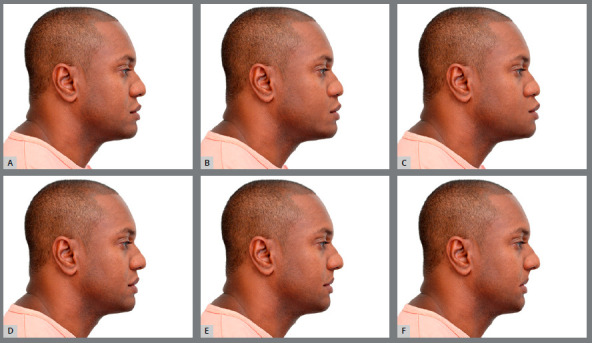
Images of the male profile: A) advancement of the lips and chin by 2mm, in relation to the true vertical line ( TVL ), B) advancement of 6mm, C) advancement of 10mm, D) retrusion of 2mm, E) retrusion of 6mm, F) retrusion of 10mm.

**Figure 2:**
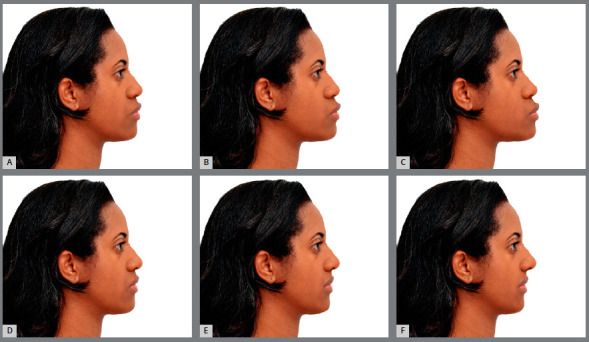
Images of the female profile: A) advancement of the lips and chin by 2mm, in relation to the true vertical line ( TVL ), B) advancement of 6mm, C) advancement of 10mm, D) retrusion of 2mm, E) retrusion of 6mm, F) retrusion of 10mm.

The initial photographic image was altered in relation to the TVL, producing changes of -2, -6, -10, +2, +6, +10mm in the upper and lower lips and chin, using the Adobe Photoshop CC (Adobe Systems, San Jose, California, 2017), without making any changes in the vertical dimension. Movements backwards from the TVL had negative values, and movements forward from the TVL had positive values. In total, six altered images were created, as shown in Figures 1 and 2. The order for displaying each photograph was randomly defined, for application of the test.^16^ ^,^ ^17^

### PROFILE PLEASANTNESS ASSESSMENT

Charts with a Visual Analogue Scale (VAS) for each photograph were distributed among the evaluators. The VAS used was 10 cm long, varying from 0 (UNPLEASANT) to 100 mm (PLEASANT). Each evaluation was made by measuring the distance, in millimeters, from 0 (UNPLEASANT) to the mark made by the evaluator.^18^ ^-^ ^20^ The photographs were projected on a 13.3-in portable LCD screen. The evaluators were initially asked to analyze the side view photographs, one by one, each photo for 15 seconds, without the possibility of going back.^19^ ^,^ ^21^ ^,^ ^22^ Afterwards, they were asked to analyze all the images simultaneously, and select the most pleasant and the most unpleasant profile, for an estimated time of 30 seconds, for each sex.

### STATISTICAL ANALYSIS

Initially, descriptive analysis of the age and sex of the evaluators was performed. Exploratory analysis of the perception and satisfaction score data was made, indicating that the data did not meet the presuppositions of a parametric analysis. Therefore, data were analyzed by generalized linear models, considering that all the observers evaluated all the images. Analysis of the images chosen as being more pleasant or more unpleasant was performed by the Fisher’s exact test. The analyses were performed with the R program, considering the level of significance of 5%.

## RESULTS

Table 1 shows that 71.7% of the evaluators were of the female sex, and the mean age of the sample was 36.7 years, with a standard deviation of 11.3 years. Table 2 shows the comparisons between the groups of evaluators, relative to the perception and satisfaction scores attributed to each photographic image. For the images of the man, the orthodontists attributed higher scores to the image with the change of -2mm in relation to the true vertical line, which did not differ significantly only from the image with the change of + 6mm. The black laypersons did not distinguish the changes of +2mm, +6mm, -2mm and -6 mm (*p*>0.05), these being the images with the highest scores. Moreover, for the male, the orthodontists and black laypersons attributed lower scores to the profiles with +10 and -10mm, without significant difference between these two images (*p*>0.05).

**Table 1:** Descriptive analysis of the age and sex of the evaluators.

Evaluator	Sex		Age (years) Mean (standard deviation)
	Female n (%)	Male n (%)	
Orthodontists	51 (73.9%)	18 (26.1%)	40.0 (10.2)
Black laypersons	48 (69.6%)	21 (30.4%)	31.3 (10.6)
General	99 (71.7%)	39 (28.3%)	36.7 (11.3)

**Table 2:** Mean (standard deviation) of perception and satisfaction scores, considering the evaluator, sex of the patient and alterations in the image, with advancement or retrusion of the lips and chin.

Change in profile^$^ (mm)	Image of the male profile		Image of the female profile	
	Orthodontists	Black laypersons	Orthodontists	Black laypersons
- 2	72.0 (20.4)^a^	61.0 (25.0)^a^	67.2 (21.7)^a^	62.6 (28.1)^a^
- 6	51.7 (24.6)^b^	50.3 (30.2)^a^	47.2 (24.8)^b^	44.0 (28.3)^b^
- 10	14.9 (17.9)^c^	*23.6 (23.8)^b^	#32.8 (24.9)^d^	#34.5 (28.0)^c^
+ 2	49.3 (25.5)^b^	50.4 (28.6)^a^	45.0 (20.7)^bc^	46.3 (28.0)^b^
+ 6	58.6 (24.2)^ab^	54.7 (28.5)^a^	#36.9 (21.3)^cd^	44.3 (28.0)^b^
+10	19.8 (21.5)^c^	20.6 (21.6)^b^	16.8 (15.7)^e^	*24.0 (23.3)^d^

Means followed by different letters in the vertical differ between them (p≤0.05). #Differ from the image of the man in the same conditions of evaluator and change (p≤0.05). *Differ from orthodontists for images of the same sex and change (p≤0.05). $In relation to the true vertical line. P (image)<0.0001; p (sex of image)=0.1927; p (evaluator)=0.0374; p (image x sex)<0.0001; p (image x evaluator)=0.0895; p (sex x evaluator)=0.5219; p (image x sex x evaluator)=0.0816.

For the woman, both orthodontists and blacks attributed higher scores to the change of +2mm, and lower scores to the change of +10mm. The orthodontists attributed significantly lower scores than the black laypersons (*p*<0.05) to the male patient with change of -10mm, and to the female patient with change of +10mm. The orthodontists also attributed lower scores to the woman than to the man (*p*<0.05) for the change of +6mm. The two groups attributed significantly lower scores to the man (*p*<0.05) for the change of -10mm.

**Figure 3:**
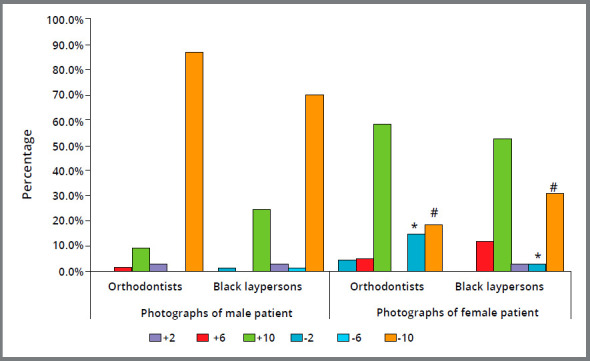
Percentage of evaluators that chose the photo as being the most unpleasant considering the Group of Evaluators and the sex of the patient, for the images changed ( in mm ) in relation to the true vertical line. *Comparison between orthodontists and black laypersons was statistically significant for the image with -6mm change. #Comparison between orthodontists and black laypersons was statistically significant for the image with -10mm change.

Figure 3 shows the frequencies and percentages of indication of each image as being the most pleasant or most unpleasant in the images analyzed together for each sex. For the man, there was no distinction between the groups of orthodontists and black laypersons, relative to the scores pleasant and unpleasant (*p*>0.05): the most pleasant profile was the image with the change of -2mm, and the most unpleasant was the -10mm. For the images of the woman, there was also no distinction between the groups (p>0.05) relative to the most pleasant image, being the most preferred the -2mm change. The image elected as being the most unpleasant of the woman was the +10mm change; however, the -6mm was considered the most unpleasant by 14.5% of the orthodontists and by only 2.9% of the blacks. The image with the change of -10mm was considered unpleasant by 30.4% of the black laypersons and by only 18.8% of the orthodontists (*p*<0.05).

## DISCUSSION

Orthodontists must be aware of the aesthetic patterns of their patients, since the parameters of beauty of each of them may be different.^22^ This is a great challenge faced by orthodontists who prefer to establish an adequate relation of occlusion and satisfactory facial aesthetics, while their patients frequently prioritize the best aesthetic results and a well-balanced appearance on conclusion of treatment.^23^ ^,^ ^24^ Due to these differences, when professionals evaluate their patients’ profile, they can decide about a treatment plan that will satisfy their patients.^17^ In a study that compared the differences between laypersons and orthodontists in assessing the correlation between attractiveness and facial components, it was found that the contribution of teeth to facial attractiveness was significantly smaller than that of lips and chin. The laypersons were more influenced by the chin and the orthodontists, by the lips. Thus, orthodontists who change the position of the teeth without worrying about the aesthetics of the lips and chin will have limited results in improving the facial attractiveness of their patients.^24^

This study was conducted to evaluate the perception of facial aesthetics among orthodontists and black laypersons in the Brazilian Northeast, a reference region for this black population,^6^ ^,^ ^10^ consisting mostly of non-white people^25^, who historically have a convex and bi-protrusion profile as the most common features among them.^26^

Profile photographs are often used by orthodontists to make the diagnosis, showing the structures of the face in detail, and therefore allow facial attractiveness to be assessed.^7^ In the present study, the majority of the evaluators were of the female sex; however, attractiveness is perceived in the same manner, irrespective of the observer’s sex.^27^ Moreover, in the present study, the authors sought to obtain an evaluation of black patients made by black laypersons, considering that it is important for orthodontists to consider the real expectations of blacks themselves, regarding the result of their facial aesthetic appearance on conclusion of orthodontic treatment. 

In the present study, there was no difference between the perception of orthodontists and black laypersons in the evaluation of the facial profile aesthetics. The two groups held a similar opinion in the classification of the most pleasant and most unpleasant profile of black individuals. This data allows professionals to have confidence in the facial aesthetic objectives established in orthodontic planning, since the pattern considered ideal was the same in the opinion of professionals and laypersons.^17^ ^,^ ^28^ ^,^ ^29^ ^,^ ^30^ It is worth emphasizing that the criteria of aesthetic appreciation are subjective.^31^

The scores attributed by the evaluators demonstrated that when the orthodontists evaluated the male profile, they were more pleased with the changes of -2mm and +6mm in relation to the true vertical line, simulating one profile considered straight and the other with slight convexity of the soft tissues, respectively. They considered that the changes of +10mm and -10mm, simulating bimaxillary protrusion and bimaxillary retrusion, respectively, were more unpleasant - the latter photographs were also classified by the blacks as being more unpleasant for the male profile. These results suggested a Class I skeletal pattern preference, particularly for black men.^1^ ^,^ ^32^ When Sena et al.^19^ evaluated the anteroposterior position of the mandible, they perceived the strong influence it exerts on the level of facial attractiveness and social perception, to the point of suggesting that having a attractive profile would also help one being hired for jobs with better economic conditions.

In the simultaneous evaluation of all the changes produced in this research, divided by sex, it was verified that the orthodontists classified the most concave (-10mm) profile for the man, and the most convex (+10mm) for the woman as being the most unpleasant profiles. In addition to the most concave profile, the black laypersons also pointed out the most convex profile as being less attractive for both sexes evaluated. This behavior demonstrated that the orthodontists more readily accepted the convex pattern when it was present in a male profile. Moreover, it revealed that this condition was not pleasing to black laypersons in either sex, particularly in the more convex female profile. In a similar manner to the studies of Almeida et al.^28^ and Oliveira et al.,^1^ the orthodontists and laypersons agreed that the skeletal discrepancies simulating Class III, represented by the more concave profile, were classified as being the worst, from an aesthetic point of view.

In this study, the straight profile with -2mm change in relation to the true vertical line was the face elected as the most attractive type by both orthodontists and black laypersons; and this profile is considered non-standard in the subjective facial analysis in both sexes evaluated. This result was compatible with that of studies in which orthodontists and laypersons preferred a slightly convex or straight profile as the most pleasant for black persons, for both African-Americans^33^ and Afro-Brazilians.^6^ The profile selected represented a less convex pattern than the type that was considered normal for black individuals, suggesting that the objective of the treatment should consider the ethnic characteristics and the point of view of the patient being treated.^33^ ^,^ ^6^ Hall et al.^17^ found a preference for increased convexity of the soft tissues for Afro-Americans, when compared with White-Americans.

The media are some of the main sources of influence for defining cultural patterns. Television films, magazines, books, and newspapers offer daily reinforcement of facial stereotypes. The profile chosen in this research appears to simulate the profiles of black movie stars,^33^ who have straight profiles that are closer to the standard patterns for whites. These celebrities have a strong influence on society because they are considered the standard of beauty, and this is reflected in people’s preferences. This factor may have influenced the perceptions expressed by both the laypersons and orthodontists (who are still mostly white) when electing the straight profile as the ideal, which is commonly seen in actors considered the most beautiful. Another important factor in determining the pattern of individual normality would be the ancestry, which can differentiate the normal from the ideal facial profile. The different characteristics of the samples and the cultural influences certainly determine one of the limitations of this type of cross-sectional observational study. 

Therefore, the treatment plan must be prepared individually and the opinion of the patient undergoing treatment must be considered more relevant than the opinion of the professional, when deciding about the most pleasant profile, considering the differences in aesthetics perception.^6^ ^,^ ^34^

## CONCLUSION

The influence of the aesthetic perception of orthodontists and black laypersons in the evaluation of the anteroposterior position of the soft tissues (lips and chin) on the facial profile of black patients was similar: The extremely concave profile (-10mm) was considered the worst and the straight profile (-2mm), the best from an aesthetic point of view, for both sexes.

Black patients, like everyone else, expect to have more beautiful faces at the end of orthodontic treatment. Therefore, the aesthetic perception must be considered in the treatment plan, and will influence the decision about the treatment strategy to be carried out by the orthodontist.
